# A Hybrid Piezoelectric and Electromagnetic Broadband Harvester with Double Cantilever Beams

**DOI:** 10.3390/mi14020240

**Published:** 2023-01-18

**Authors:** Bing Jiang, Fan Zhu, Yi Yang, Jingyu Zhu, Yuting Yang, Ming Yuan

**Affiliations:** 1 College of Automation & College of Artificial Intelligence, Nanjing University of Posts and Telecommunications, Nanjing 210023, China; 2 Key Laboratory of Architectural Acoustic Environment of Anhui Higher Education Institutes, Hefei 230601, China

**Keywords:** vibration energy harvester, double cantilever beams, piezoelectric, electromagnetic, hybrid harvester

## Abstract

Vibration-energy harvesting is an effective strategy for replacing batteries and provides a long-term power supply to microelectronic devices. Harvesting vibration energy from human motions has attracted research attention in recent years. Here, a novel low-frequency hybrid piezoelectric and electromagnetic broadband harvester is proposed. Two parallel piezoelectric cantilever beams support the harvester and capture environmental vibration energy based on the piezoelectric effect. A permanent magnet is connected by springs to the two beams, and a fixed coil surrounds the moving permanent magnet, enabling energy conversion via the electromagnetic effect and the proof mass. The parameters influencing the output power of the harvester are optimized numerically to boost the harvester’s performance. The output power of the proposed hybrid harvester is compared with that of a piezoelectric harvester and an electromagnetic harvester. The simulation results show that the output power is significantly higher for the hybrid harvester than for the piezoelectric and electromagnetic harvesters, and the bandwidth is broader owing to the double cantilevers. An experiment is conducted using a prototype of the hybrid harvester to evaluate its output power. The results show multiple resonant peaks, an extended bandwidth, and a maximum power of 6.28 mW. In contrast, the maximum harvested power of the piezoelectric harvester is only 5.15 mW at 9.6 Hz.

## 1. Introduction

In recent years, microelectronic devices and wireless sensor nodes have become ubiquitous in our daily life. However, the lack of a long-term stable power supply has limited the use of these devices. Chemical batteries and lithium batteries have shortcomings [1,2,3,4,5,6,7,8,9,10,11,12,13,14,15]. In contrast, mechanical vibration-energy harvesting represents a suitable alternative. Electromagnetic energy-harvesting devices harvest ambient energy based on electromagnetic induction, providing high output current and low output voltage [16]. Piezoelectric energy-harvesting devices utilize the piezoelectric effect to harvest ambient energy, producing high output voltage, high-power density, and low output current; however, they are sensitive to the ambient resonant frequency [17,18,19,20,21,22]. Therefore, hybrid vibration energy harvesters have been investigated to combine the advantages of piezoelectric and electromagnetic energy-harvesting devices.

For example, Lin et al. proposed a hybrid vibration power generator based on piezoelectric and electromagnetic mechanisms. It consisted of a 36 mm × 17 mm × 0.73 mm piezoelectric cantilever and a 40 mm long electromagnetic tube. The generator produced a high power output using the piezoelectric effect and electromagnetic induction under low-frequency vibrations. The power output (2.173 mW at 25 Hz of 1 g) was higher than that of generators with the same dimensions and conventional designs [23]. Li et al. proposed a galloping piezoelectric-electromagnetic energy harvester (GPEEH) to supply energy to low-power microelectronic devices on-site. It consisted of a galloping piezoelectric energy harvester (GPEH) and an electromagnetic energy harvester (EEH) installed inside the bluff body of the GPEH. The experimental results showed that the effective output power of the GPEEH was 112.5% higher than that of the classical galloping piezoelectric energy harvester (CGPEH) [24]. Challa et al. proposed coupling two independent energy-harvesting techniques to provide higher electrical damping in the system. The coupled energy-harvesting device consists of a primary piezoelectric energy-harvesting device to which an electromagnetic component is added to better match the total electrical damping to the mechanical damping in the system. The first coupled device has a resonance frequency of 21.6 Hz. It generates a peak power output of 332 μW, compared to 257 and 244 μW obtained from the optimized, stand-alone piezoelectric and electromagnetic energy-harvesting devices, resulting in a 30% increase in power output. A second coupled device, which utilizes the d_33_ piezoelectric mode, has a 65% higher power output than the stand-alone, single-harvesting mode devices [25]. Pyo et al. proposed a hybrid energy harvester with frequency up-conversion structures. The harvester comprises a flexible substrate and two (internal and external) cantilevers. The separate internal and external cantilevers for piezoelectric and electromagnetic transduction enable the piezoelectric internal cantilever to generate a high output power with large displacement vibration. The maximum output power of the hybrid harvester is 7.38 mW, with outputs of 1.35 and 6.03 mW for piezoelectric and electromagnetic conversion, respectively [26]. Foisal et al. designed and fabricated an array of four generators. In model A, four individual generators were placed side by side, whereas in model B the generators were placed one above the other. The experimental results showed that the power of model A and model B was 21.92 μW /cm^3^ and 52.02 μW /cm^3^, respectively, at an acceleration of 0.5 g [27]. Ganapathy et al. designed and optimized a magnetically-tunable hybrid piezoelectric-triboelectric energy harvester (MT-HPTEH). Output power of 659 µW was obtained at 180 kΩ and 44 Hz from the optimized MT-HPTEH, with a theoretical-experimental discrepancy of less than 10%. The magnetic tunability enables the harvester to work at the desired frequency range from 38 Hz to 54 Hz with an open-circuit voltage ranging from 7.8 V to 20.314 V [28]. Zhu et al. designed and analyzed a magnetoelectric energy harvester that uses Terfenol-D/PZT/Terfenol-D laminate to harvest energy from nonlinear vibrations created by magnetic levitation. Due to the high energy density and strong magneto-mechanical coupling effect of the magnetostrictive material, the proposed harvester can generate very high voltage and power at low-frequency ranges [29]. Cao et al. researched the design and tested the output performance of a double-end clamped microelectromechanical system (MEMS)-coupled piezoelectric–electromagnetic energy harvester. The result showed that the capacity of this energy harvester was 12.23 times higher than that of a piezoelectric energy harvester (PEH) [30]. Low-frequency energy harvesters that harvest energy from human motion have been investigated. Rawnak et al. presented the WE-harvest system, a wearable energy harvesting system that combines piezoelectric and electromagnetic energy harvesters in one unit to generate electrical energy. Several power conditioning circuit topologies have been proposed for efficient energy extraction from the two sources. Experimental results have demonstrated that the combined topology enhances the power generation efficiency and enables stable DC output voltages [31]. Izadgoshasb et al. improved the efficiency of a PEH for obtaining energy from human motion using a double pendulum system coupled with magnetic interaction. Three PEH configurations were investigated: a conventional PEH with a cantilever beam (PEHCB), a PEH with a single pendulum system (PEHSP), and a PEH with a double pendulum system (PEHDP). The results demonstrated that the proposed PEHDP generated multiple impacts in each motion cycle and produced higher voltage and power than the conventional PEHCB [32]. Song et al. proposed a novel piezoelectric-electromagnetic hybrid vibration energy harvester (HVEH). As the magnets moved back and forth, the piezoelectric vibration-energy harvester (PVEH) generated stable output energy. A closed magnetic circuit was designed for an electromagnetic vibration energy harvester (EVEH) with a pair of magnets and a soft magnetic core. The experimental results showed that the optimal load resistance and the maximum output power of the PVEH were 398.7 kΩ and 87.9 μW, whereas that of the EVEH was 3.2 kΩ and 2.173 mW, respectively, in cycle experiments with a frequency of 5 Hz [33]. In addition, hybrid broadband vibration-energy harvesters have been developed and analyzed. He et al. investigated a low-frequency hybrid piezoelectric-electromagnetic-triboelectric broadband vibration-energy harvester. It consists of flexible piezoelectric-electromagnetic-triboelectric picking-up vibration structures to harvest broadband vibrations at low acceleration and at a wide vibration frequency. The piezoelectric, electromagnetic, and triboelectric EH units reach 3.5 Hz, 10.0 Hz, and 18.1 Hz operating bandwidth, respectively, under 0.5 g acceleration at 20 Hz [34]. The utilization of springs in energy harvesters has also been investigated. Aldawood et al. designed an improved magnetic spring-based energy harvester that uses a dual-mass spring and a nonlinear mechanical planar spring to improve the power metrics of traditional magnetic spring-based energy harvesters significantly. The improved harvester generates 1.97 mW/cm^3^g^2^ at 0.4 g [35]. Febbo et al. developed a rotational power scavenging system as an alternative to cantilever beams attached to a hub. A versatile geometric configuration with two elastic beams and two heavy masses joined by a spring was proposed. The output power of a simple harvesting circuit, which served as an energy storage device, was in the range of 26–105 μW over the whole frequency range [36]. In addition, extensive research has recently been conducted on the static bending and free vibration responses of piezoelectric nanobeams, which is helpful in designing piezoelectric beam structures for practical applications. Doan et al. studied the mechanical behavior of the nanoplates under flexoelectric effects. This was the first study that examined the vibration response and static buckling of variable flexoelectric nanoplates using the finite element method (FEM) and novel shear deformation theory type hyperbolic sine functions [37]. Nguyen et al. combined the FEM with a novel third-order shear deformation beam theory (TSDT) to simulate the static bending and free vibration responses of rotating (around one fixed axis) piezoelectric nanobeams with geometrical imperfection, considering flexoelectric effects. The structures were placed on Pasternak’s elastic foundations. The results are highly applicable to the design of nanobeam structures in practice [38]. Phung et al. studied the rectangular plates subjected to static loads and supported on a discontinuous two-parameter elastic foundation. The formulae for the computations were developed from improved shear deformation theory. A parameter study was carried out to capture the effect of some material and geometrical parameters on the static response of structures [39]. Le et al. used the FEM to simulate the mechanical, electric, and polarization behaviors of piezoelectric nanoplates resting on elastic foundations subjected to static loads, considering the flexoelectric effect. The numerical results showed that the flexoelectric effect significantly affected the mechanical responses of the nanoplates [40].

In summary, substantial achievements have been made in researching piezoelectric and electromagnetic energy-harvesting devices. However, the following limitations/problems remain. The harvested power is still lower than that of conventional power sources. The resonant frequencies of most harvesters are higher than the frequency band of human movement. The effective energy-harvesting bandwidth is narrow, resulting in low harvesting efficiency. In addition, the piezoelectric material of PVEHs has low durability.

This paper proposes a novel hybrid piezoelectric and electromagnetic broadband harvester (HPEBH). Two kinds of energy harvesters (piezoelectric and electromagnetic energy harvesters) are combined to improve the output power. Two piezoelectric cantilever beams are placed in parallel to generate two resonance peaks in the first-order vibration mode. The frequency range covered by the two resonance peaks is much broader than the effective energy-harvesting bandwidth of the single-beam structure. A permanent magnet acts as the proof mass at the end of the beams, lowering the energy harvesting bandwidth to the frequency band of human movement. Two springs connect the two cantilever beams with the permanent magnet and act as the support and guide of the moving magnet. The springs constrain the permanent magnet’s vibration path so that the fixed coil can be placed close to the permanent magnet, resulting in higher harvesting efficiency of the electromagnetic harvester. The springs contribute to energy storage and conversion, reducing the sudden impact of environmental vibration. As a result, the springs improve the durability of the piezoelectric cantilever beams. Finite element simulation analysis and verification experiments are performed to demonstrate the ability of the HPEBH to harvest energy from human movement.

The structure of this paper is organized as follows: Section 1 briefly reviews the related works describing existing problems and the primary purpose of this work. Section 2 presents the mathematical model of the HPEBH. Section 3 describes the numerical optimization of the parameters influencing the harvester’s output power. The experimental results and discussion are presented in Section 4, and Section 5 concludes the paper.

## 2. HPEBH Structure and Mathematical Model

**Figure 1:**
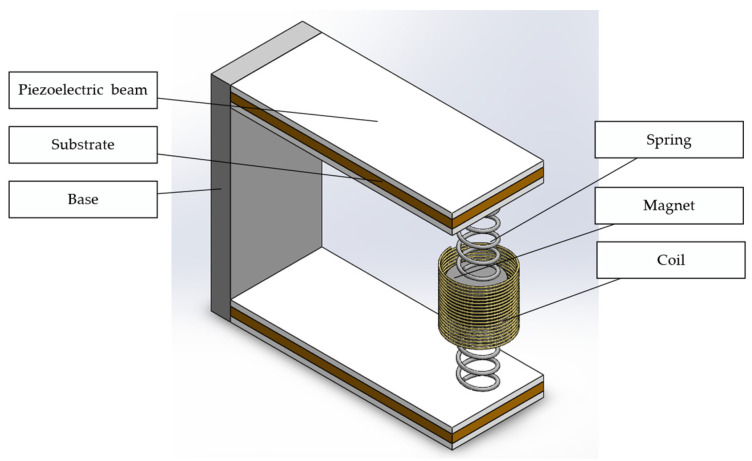
Hybrid piezoelectric and electromagnetic broadband harvester (HPEBH) structure.

The proposed HPEBH has two parallel piezoelectric cantilever beams. The ends of the beams are connected to a permanent magnet by springs. A fixed coil surrounds the permanent magnet to harvest vibration energy, as shown in Figure 1.

**Figure 2:**
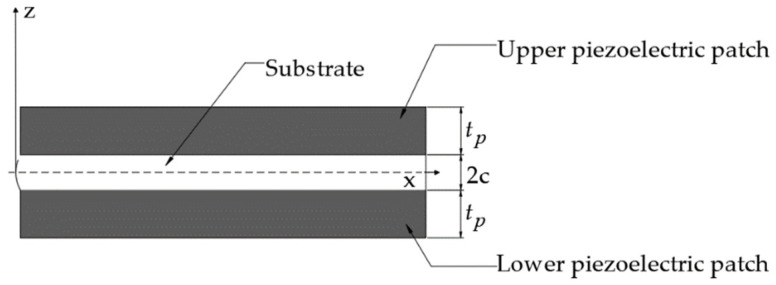
The cross-sectional structure of the piezoelectric cantilever beam.

The cantilever beam consists of an elastic metal substrate (the material is beryllium bronze), piezoelectric ceramic patches (the material is PZT-5H), end springs, and a proof mass (permanent magnet). Figure 2 shows the cross-section of the piezoelectric cantilever beam. The thickness of the upper and lower piezoelectric patches is *t_p_*, and that of the elastic beam is 2*c*. The width of the piezoelectric patch is *b*, and the length of the piezoelectric cantilever beam is *L_p_*. The permanent magnet acts as the proof mass of the piezoelectric harvester, lowering the resonant frequency of the energy-harvesting structure. It also serves as the mover of the electromagnetic harvester. The motion of the permanent magnet generates an induced electromotive force in the fixed coil. The piezoelectric harvester and electromagnetic harvester are connected to the load resistances *R_L1_* and *R_L2_*, which match their internal resistance values.

The advantages of the HPEBH structure are as follows: (1) Two kinds of energy harvesters (piezoelectric and electromagnetic energy harvesters) are combined to improve the output power. (2) The double-beam structure generates two first-order resonance peaks in the low-frequency band, increasing the bandwidth of the energy harvester. (3) The springs constrain the permanent magnet’s vibration path; thus, the fixed coil can be placed close to the permanent magnet, improving the electromagnetic harvester’s energy-harvesting efficiency. (4) The springs reduce the sudden impact of environmental vibration and improve the durability of the piezoelectric cantilever beams.

**Figure 3:**
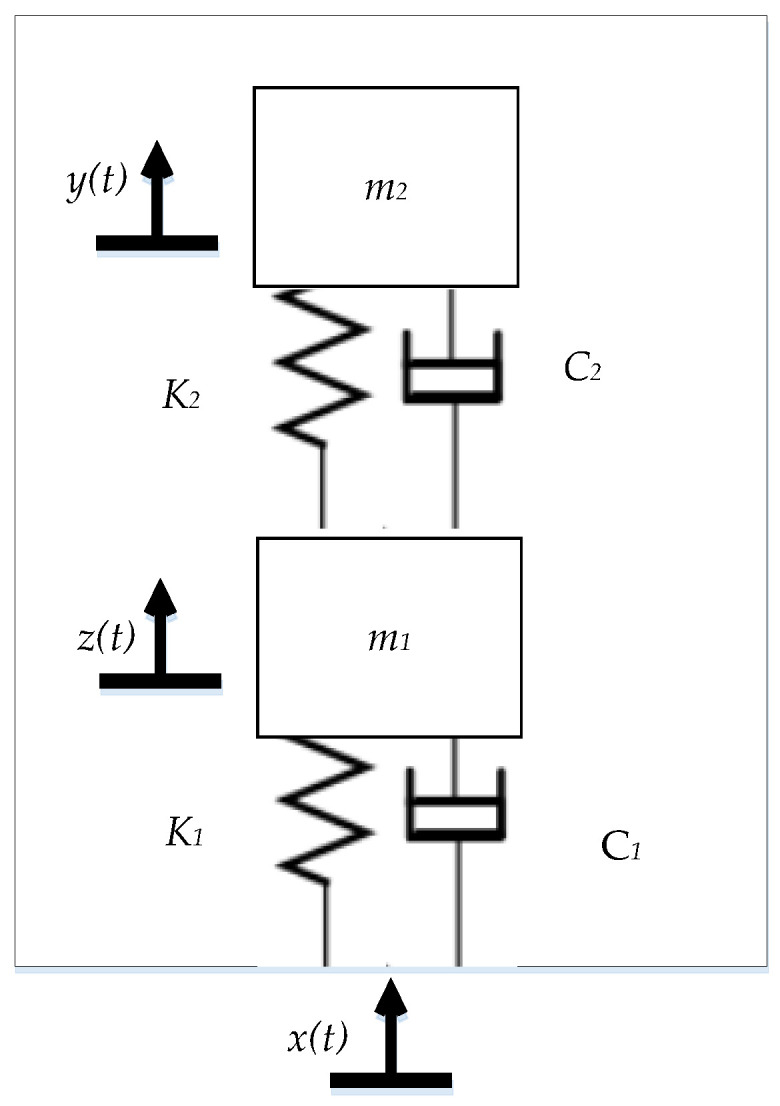
HPEBH model with elastic amplification.

The HPEBH’s mathematical model can be simplified to a spring-mass-damper system, as shown in Figure 3.

In Figure 3, $C_{1}$ and $C_{2}$ represent the damping of the piezoelectric harvester and the electromagnetic harvester, respectively. $C_{1}$ includes the piezoelectric damping $C_{1P}$ and the mechanical damping $C_{1m}$ of the piezoelectric cantilever beams. $C_{2}$ of the electromagnetic harvester includes the springs’ mechanical damping $C_{2m}$ and electromagnetic damping $C_{2e}$.$\;K_{1}$ represents the piezoelectric cantilever beam’s equivalent stiffness, and $K_{2}$ represents the springs’ equivalent stiffness. *m*_1_ represents the piezoelectric cantilever beam’s equivalent mass, and *m*_2_ represents the magnet’s equivalent mass. x(t) is the vibration exciter’s input vibration, z(t) is the displacement of the piezoelectric cantilever beam’s equivalent mass, and y(t) is the equivalent displacement of the magnet proof mass.

The differential equations of motion of the system are defined as follows: [12,41]

(1) $$m_{1}\ddot{z}\left(t\right)+c_{1}\dot{z}\left(t\right)+K_{1}z\left(t\right)=c_{1}\dot{x}\left(t\right)+K_{1}x\left(t\right)$$

(2) $$m_{2}\ddot{y}\left(t\right)+c_{2}\dot{y}\left(t\right)+K_{2}y\left(t\right)=c_{2}\dot{x}\left(t\right)+K_{2}x\left(t\right)$$

Based on the coupled piezoelectric and electromechanical properties, the voltage generated on the surface of a piezoelectric material is correlated to the stress applied to the material [25].

(3) $$V=\frac{2d_{31}t_{p}\delta_{avg}}{\varepsilon }$$

where $t_{p}$ represents the thickness of the piezoelectric ceramic patch (m); $d_{31}$ represents the piezoelectric constant (C/N); $\delta_{avg}$ represents the average stress on the piezoelectric cantilever beam (Pa); and ε represents the dielectric constant of the piezoelectric ceramic patch (F/m). 

The internal resistance of the piezoelectric material is approximately equivalent to the capacitive reactance, and its value is $R_{p}=\frac{t_{p}}{\pi f\varepsilon bL_{p}}$, where *f* represents the vibration frequency of the piezoelectric cantilever beam. If the load resistance is $R_{L1}$, the output power of the piezoelectric harvester is:

(4) $$P_{p}=\frac{\alpha^{2}R_{L1}Z_{0}}{{\left(R_{P}+R_{L1}\right)}^{2}}$$

where α is the electromechanical coupling coefficient, and $Z_{0}$ refers to the root mean square value of z(t). Equation (4) indicates that the maximum output power of the piezoelectric harvester occurs at the optimal load resistance, which is proportional to the vibration displacement.

Based on the law of electromagnetic induction, if the cross-sectional areas of the coils are the same, the induced electromotive force can be expressed as follows: [27]

(5) $$E=-\frac{d\Phi }{dt}=-N\frac{d\varphi }{dt}$$

where *N* represents the number of coil turns; Φ refers to the total magnetic flux linkage passing through the coils; and φ refers to the magnetic flux linkage passing through a single coil. The axial magnetic flux density *B* of the cylindrical permanent magnet may be expressed as follows:

(6) $$B=\frac{B_{r}}{2}\left[\frac{h}{\sqrt{h^{2}+r^{2}}}+\frac{h-h_{m}}{\sqrt{{\left(h-h_{m}\right)}^{2}+r^{2}}}\right]$$

where $B_{r}$ represents the residual magnetic flux density of the permanent magnet; $h_{m}$ is the height of the permanent magnet; r is the radius of the permanent magnet; and h is the distance from the coil to the bottom surface of the permanent magnet. The following equation can be derived from Equation (6):

(7) $$B_{h}=\frac{dB}{dh}=\frac{B_{r}r^{2}}{2}\left[\frac{1}{{\left(h^{2}+r^{2}\right)}^{3/2}}+\frac{1}{{\left[{\left(h-h_{m}\right)}^{2}+r^{2}\right]}^{3/2}}\right]$$

Equation (5) can be changed as follows:

(8) $$E=-NS\frac{dB}{dh}\frac{dh}{dt}=NSB_{h}\dot{H\left(t\right)}=\beta \dot{H\left(t\right)}$$

where $\dot{H\left(t\right)}$ represents the velocity of the magnet relative to the fixed end, i.e.,$\dot{\;H\left(t\right)}=\dot{y\left(t\right)}-\dot{x\left(t\right)}$. β represents the equivalent electromagnetic coupling coefficient,$\;\beta =NSB_{h}$.

When the permanent magnet at the end of the piezoelectric cantilever beam moves, its output power is defined as follows:

(9) $$P_{em}=\frac{\beta^{2}R_{L2}\dot{H_{0}}}{{\left(R_{c}+R_{L2}\right)}^{2}}$$

where $R_{c}$ represents the coil’s internal resistance; $R_{L2}$ represents the load resistance of the electromagnetic harvester; and $\dot{H_{0}}$ represents the root mean square value of $\dot{H\left(t\right)}$. Equation (9) indicates that the electromagnetic harvester has an optimal load resistance, and the output power is correlated to the movement velocity of the permanent magnet. 

## 3. Finite Element Simulation

The FEM simulation flow is as follows:

- A finite element simulation model of the HPEBH is established in COMSOL. The piezoelectric harvester is analyzed to obtain the resonant frequency, output voltage, optimum load resistance, and output power of the piezoelectric harvester.
- A model of an electromagnetic harvester is created in Maxwell software to analyze the harvester’s optimum load resistance and output power and obtain the electromagnetic motion’s damping force.
- The damping force obtained from the simulation of the electromagnetic harvester is used for the piezoelectric harvester to obtain its output voltage and output power in the hybrid simulation. The motion function of the end magnet is derived.
- Analysis of the electromagnetic harvester is conducted in Maxwell to obtain its output power in the hybrid simulation.

### 3.1. Simulation of Piezoelectric Energy Harvesting

PZT-5H is used as the upper and lower piezoelectric patches of the HPEBH. The patch is 80 mm long, 33 mm wide, and 0.2 mm thick. The cylindrical permanent magnet is a NdFeB magnet with a diameter of 10 mm and a height of 10 mm. The piezoelectric cantilever beam’s substrate is 100 mm long, 33 mm wide, and 0.15 mm thick. The material parameters are listed in Table 1.

Finite element modal analysis of the HPEBH is performed in COMSOL. A fixed constraint is placed at the left end of the piezoelectric cantilever beam’s substrate. The upper and lower piezoelectric cantilever beams’ right ends are connected to the magnet by springs. The spring material is phosphor bronze. The number of spring coils *n* is 12, and the wire diameter *d* and outer diameter *d_2_* of the coil are 0.4 mm and 6 mm, respectively. The elastic coefficient is 0.5 N/cm and the acceleration is 0.5 g. 

**Figure 4:**
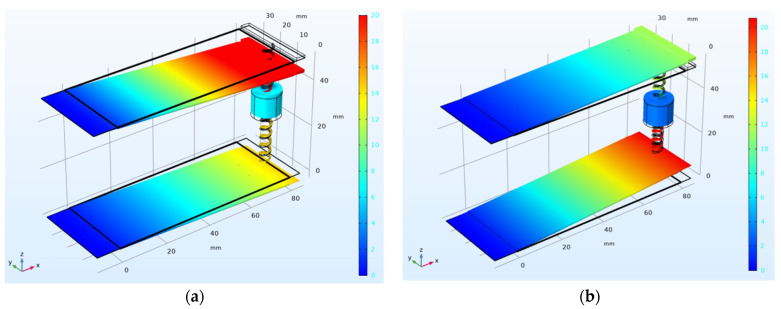
First-order vibration mode of the HPEBH: (**a**) Displacement of the first resonance point; (**b**) Displacement of the second resonance point.

As shown in Figure 4, the HPEBH generates two displacement resonance peaks in the first-order vibration mode. These are hereafter referred to as the first resonance point and the second resonance point. The displacement of the piezoelectric cantilever beam becomes more significant as the motion of the end mass increases in the first-order mode; the displacement is distributed uniformly. Moreover, the frequency is low at the first-order resonance point, facilitating energy harvesting. Meanwhile, in the first-order vibration mode, the piezoelectric cantilever beam moves up and down with the proof mass at the end of the beam, with the same movement direction as the permanent magnet in the HPEBH. Therefore, we focus on the first-order harmonic response of the HPEBH.

#### 3.1.1. Harmonic Response Analysis of the Piezoelectric Harvester

**Figure 5:**
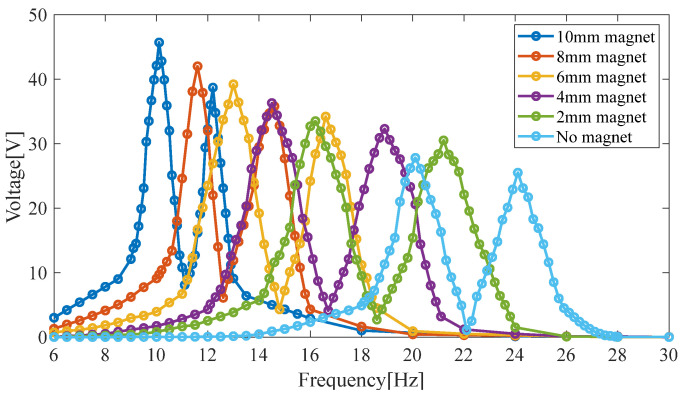
The open-circuit voltage of the piezoelectric harvester with different masses of the permanent magnet.

A sinusoidal excitation is applied to the HPEBH. The magnet blocks’ radius is 5 mm, and their heights are 2 mm, 4 mm, 6 mm, 8 mm, and 10 mm, respectively. The results of the harmonic response analysis of the piezoelectric cantilever beam’s open-circuit voltage at an acceleration of 0.5 g are shown in Figure 5. 

In Figure 5, the results show that the first-order resonant frequency of the HPEBH ranges from 5 Hz to 25 Hz. As the mass of the permanent magnet increases, the frequencies at the first and second resonance points decrease, and the peak value of the open-circuit voltage increases. For a magnet with a height of 10 mm and a radius of 5 mm, the first (second) resonance point’s frequency is 10.1 Hz (12.2 Hz), and the peak value of the open-circuit voltage is 45.7 V (38.7 V).

The larger the magnet’s mass, the greater the displacement at the end of the piezoelectric cantilever beam. This displacement is conducive to vibration-energy harvesting, although an excessive displacement may affect the stability of the piezoelectric patches. Therefore, a permanent magnet with a height of 10 mm is used as the proof mass to balance the power-generation performance of the piezoelectric harvester and the stability of the piezoelectric patches.

#### 3.1.2. Impact of the Load Resistance on the Output Power of the Piezoelectric Harvester

**Figure 6:**
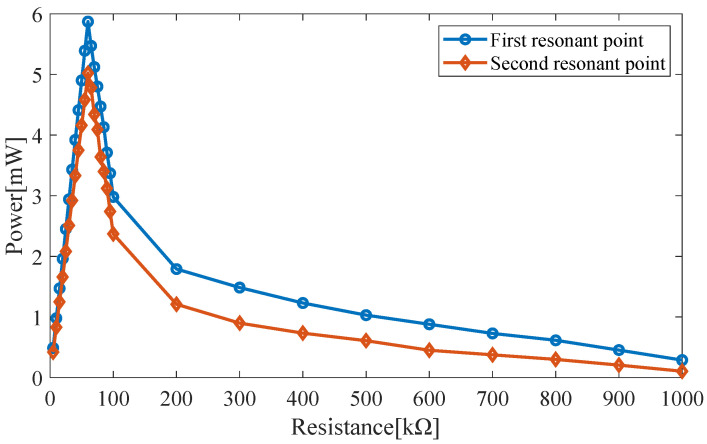
Output power for different load resistances.

Since the load resistance may affect the output power of the piezoelectric harvester, a cylindrical magnet with a height of 10 mm and a radius of 5 mm and peak resonant frequencies of 10.1 Hz and 12.2 Hz is used. The outputs at the two resonance points for different load resistances are shown in Figure 6. 

The output power at the first and second resonance points reaches its peak at the load resistance of 61 kΩ. 

#### 3.1.3. Impact of the Excitation Acceleration on the Output Power of the Piezoelectric Harvester

**Figure 7:**
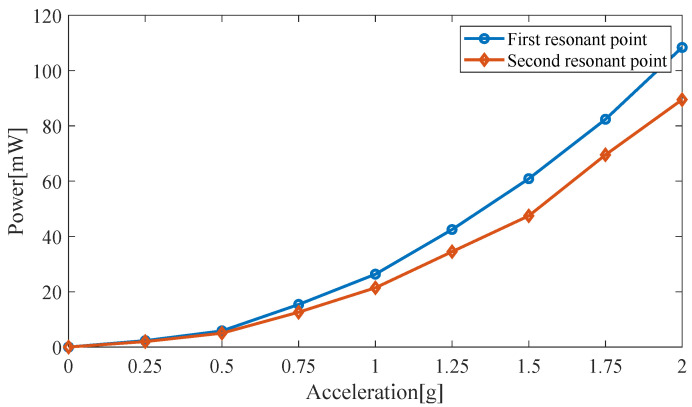
Output power for different excitation accelerations.

In this model, a cylindrical permanent magnet with a height of 10 mm and a radius of 5 mm is used; the resonant frequencies are 10.1 Hz and 12.2 Hz. The excitation acceleration is increased, and the load resistance is 61 kΩ. The effect of the excitation acceleration on the output power of the piezoelectric harvester is shown in Figure 7. The faster the excitation acceleration, the greater the amount of energy harvested, as long as the stability of piezoelectric double beams is ensured.

#### 3.1.4. Effect of the Ambient Frequency on the Amplitude of the Permanent Magnet 

**Figure 8:**
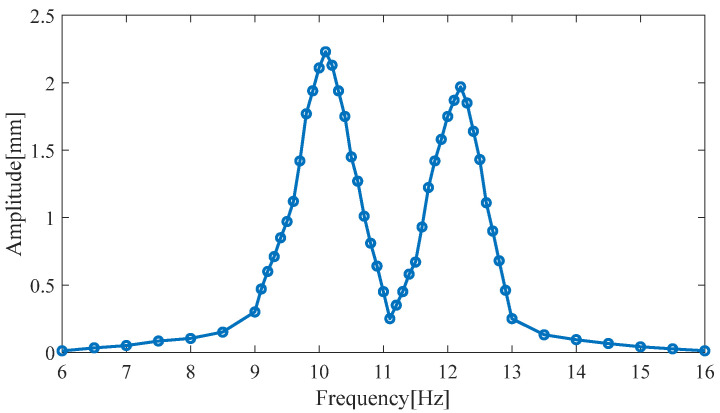
The permanent magnet’s amplitude for different ambient frequencies.

A point probe is placed on the magnet in the piezoelectric harvester in COMSOL to obtain the permanent magnet’s amplitude for different ambient frequencies, as shown in Figure 8.

The permanent magnet has a radius of 5 mm and a height of 10 mm. The amplitude of the permanent magnet reaches the first peak (2.23 mm) at a vibration frequency of 10.1 Hz (the frequency of the first resonance point) and the second peak value (1.97 mm) at a vibration frequency of 12.2 Hz (the frequency of the second resonance point).

The load resistance is 61 kΩ. The simulated output voltage of the piezoelectric harvester produces two resonance peaks in the frequency range of 9–13 Hz. The first peak value is 10.1 Hz (the first resonance point); the output voltage is 28.2 V; and the output power of the two piezoelectric cantilever beams is 5.87 mW. The second peak occurs at 12.2 Hz (the second resonance point); the output voltage is 23.9 V; and the output power is 5.02 mW.

### 3.2. Maxwell Simulation of the Electromagnetic Harvester

#### 3.2.1. Effect of Different Loads on the Power Output of the Electromagnetic Harvester

The Maxwell simulation of the electromagnetic harvester is divided into moving-iron and moving-coil simulations. The permanent magnet’s motion is derived from the vibration at the end of the piezoelectric cantilever beams. Therefore, we use a moving-iron simulation model. A cylindrical magnet (N35) with a radius of 5 mm and a height of 10 mm is used. The energy-harvesting coil material is copper, with 2000 coil turns.

**Figure 9:**
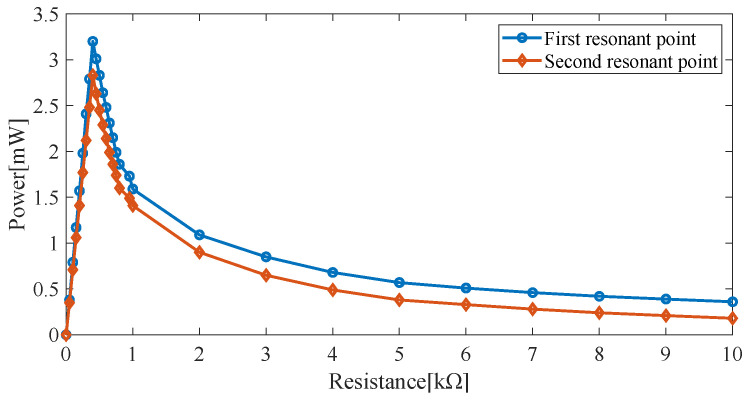
Output power of the electromagnetic harvester for different load resistances.

The output power of the electromagnetic harvester of the HPEBH for different load resistances is shown in Figure 9. The output power reaches its peak when the first and second resonance points are at the optimum load resistance of 0.4 kΩ.

#### 3.2.2. Output Power of the Electromagnetic Harvester

The trajectory of the moving magnet in the electromagnetic simulation model is a parasinusoidal motion. The piezoelectric harvester produces two resonance peaks. The simulation analysis of the electromagnetic harvester also focuses on these two resonance points. The load resistance is 0.4 kΩ. Based on the harmonic response of the piezoelectric harvester, the frequency at the first resonance point is 10.1 Hz, and the displacement amplitude of the permanent magnet is 2.23 mm. The frequency at the second resonance point is 12.2 Hz, and the displacement amplitude of the permanent magnet is 1.97 mm. The Maxwell motion simulation function is obtained from differentiation of the sine displacement function as follows:

(10) $$S_{1}\left(t\right)=0.1414\cdot \cos(63.4t)$$

(11) $$S_{2}\left(t\right)=0.1509\cdot \cos(76.6t)$$

**Figure 10:**
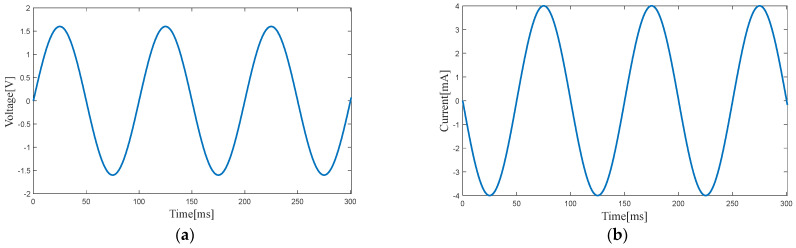
Voltage (**a**) and current (**b**) of the electromagnetic harvester at the first resonance point.

**Figure 11:**
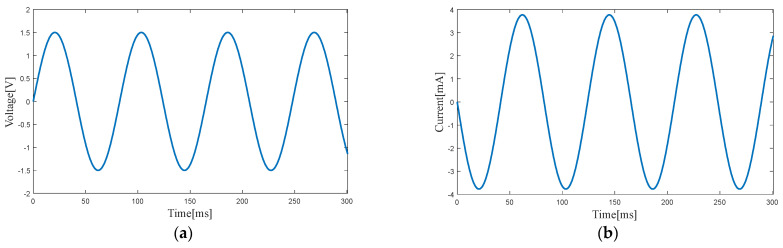
Voltage (**a**) and current (**b**) of the electromagnetic harvester at the second resonance point.

Figure 10 and Figure 11 show the output voltage and output current of the electromagnetic harvester at the two resonance points. The output power of the electromagnetic harvester is 3.20 mW at 10.1 Hz (first resonance point) and 2.83 mW at 12.2 Hz (second resonance point).

**Figure 12:**
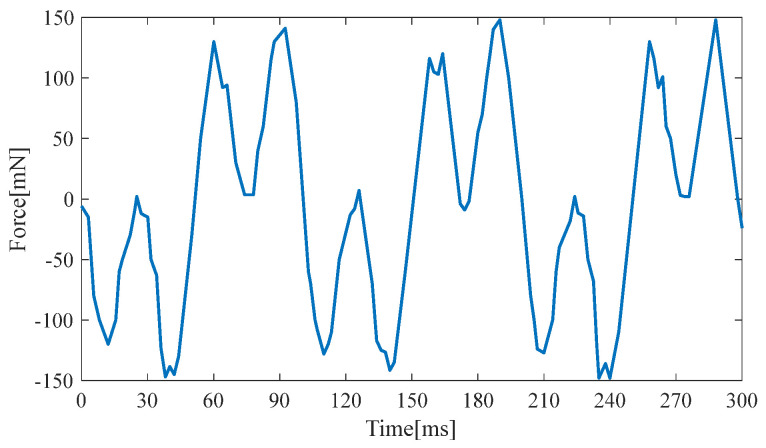
Electromagnetic damping force at the first resonance point.

**Figure 13:**
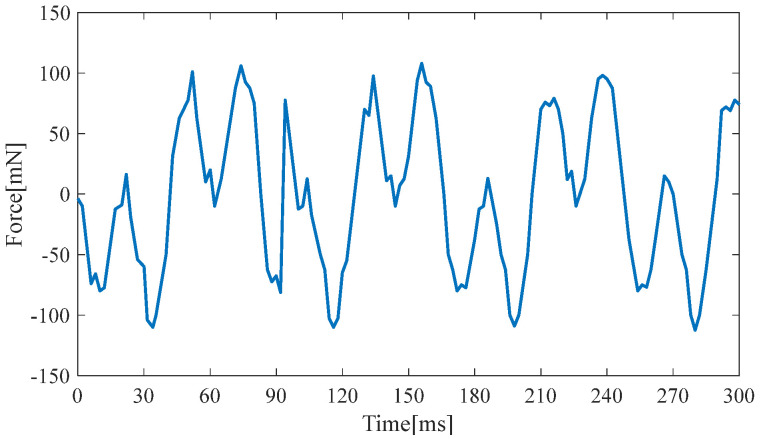
Electromagnetic damping force at the second resonance point.

Figure 12 and Figure 13 show the electromagnetic damping force generated at the two resonance points. The maximum electromagnetic resistance is 0.15 N and 0.12 N, respectively. Therefore, the approximate electromagnetic damping force equation for the two resonance points is as follows:

(12) $$F_{e1}=0.15\cdot \sin(63.4t)$$

(13) $$F_{e2}=0.12\cdot \sin(76.6t)$$

A large damping force and a high resonance point are used in the simulation to obtain a conservative estimate of the hybrid energy-harvesting performance. Thus, the damping force equation is as follows:

(14) $$F_{e12}=0.15\cdot \sin(76.6t)$$

### 3.3. Energy-Harvesting Simulation of the HPEBH

**Figure 14:**
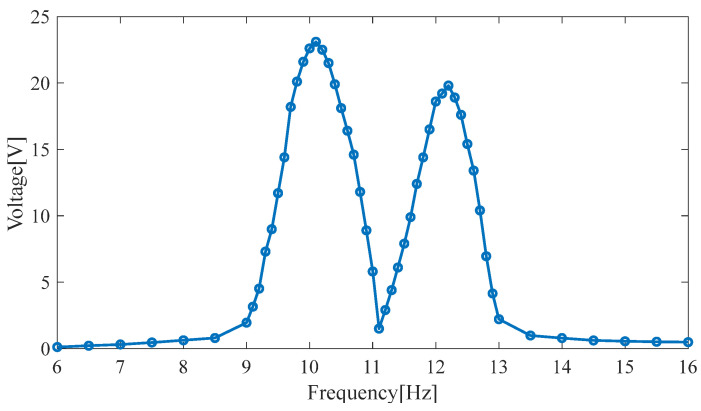
Output voltage of the HPEBH’s piezoelectric harvester.

After the electromagnetic damping force has been used in the piezoelectric harvester and the finite element simulation of the hybrid structure has been performed (Figure 14), the output voltage of the HPEBH’s piezoelectric harvester is 23.1 V at 10.1 Hz (first resonance point) and 19.8 V at 12.2 Hz (second resonance point). The electromagnetic damping force has weakened the performance of the piezoelectric harvester.

**Figure 15:**
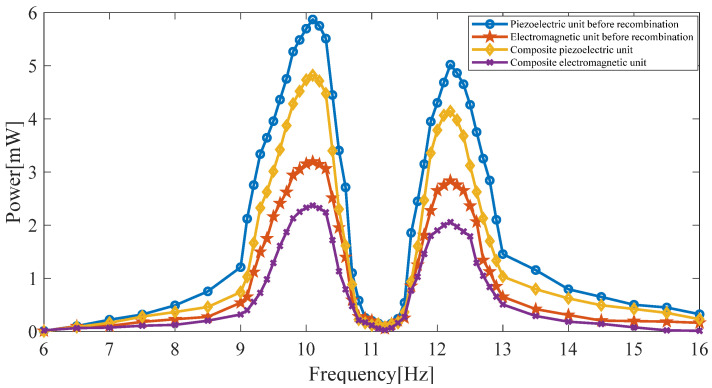
Output power of the HPEBH.

Figure 15 shows the simulated output power of the HPEBH. The output power of the HPEBH’s piezoelectric harvester is 4.82 mW at 10.1 Hz (first resonance point) and 4.14 mW at 12.2 Hz (second resonance point).

Owing to the electromagnetic damping force, the amplitude of the piezoelectric cantilever beam is lower for the HPEBH than for the piezoelectric harvester, affecting the amplitude of the moving magnet. The Maxwell motion simulation function of the permanent magnet in the HPEBH model is recalculated and used as the amplitude of the permanent magnet in the hybrid harvester. The output power of the electromagnetic harvester in the HPEBH (Figure 15) is 2.37 mW at 10.1 Hz (first resonance point) and 2.07 mW at 12.2 Hz (second resonance point).

The finite element simulation results of the HPEBH show that the output power values of the HPEBH’s piezoelectric harvester and the HPEBH’s electromagnetic harvester are lower owing to the electromagnetic damping force. However, the total output power of the HPEBH is 7.19 mW at the first resonance point, which is 22.5% higher than that of the piezoelectric harvester. The total output power of the HPEBH at the second resonance point is 6.21 mW, which is 23.7% higher than that of the piezoelectric harvester. Since the output power of the electromagnetic harvester is lower than that of the piezoelectric harvester, the total output power of the HPEBH is higher than that of the electromagnetic harvester. Since the HPEBH has a double-beam structure, the two resonance peaks broaden the frequency band. As shown in Figure 14, the effective energy-harvesting bandwidth of the piezoelectric harvester of the HPEBH is about 4 Hz, which is much higher than the effective energy-harvesting bandwidth of the harvester with one beam and is within the low-frequency band of 9–13 Hz, consistent with the frequency of human motion.

## 4. Experimental Results and Discussion

**Figure 16:**
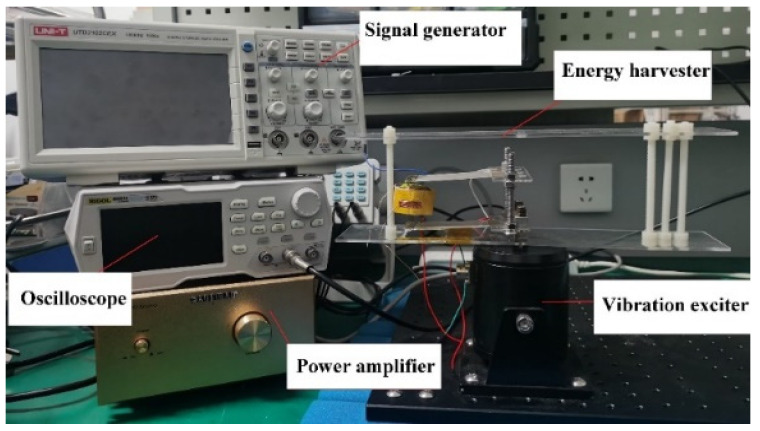
Test platform for the experiment.

The experimental system consists of an oscilloscope, a signal generator, a power amplifier, a vibration exciter, and the proposed HPEBH (Figure 16). The HPEBH parameters, motion parameters, and optimal load resistance are the same as those in the simulation. Two acrylic plates are attached to the vibration table of the vibration exciter, and the electromagnetic induction coil is sandwiched between the two acrylic plates. The ends of the two piezoelectric cantilever beams are screwed to the vibration table of the exciter. A permanent magnet is connected to the suspended ends of the two piezoelectric cantilever beams by springs. The coil generates an induced electromotive force when the permanent magnet moves with the piezoelectric cantilever beam. The signal generator generates signals at the required frequency that are conveyed to the vibration exciter through a power amplifier. The power amplifier regulates the excitation acceleration of the vibration exciter to generate vibration signals that will power the HPEBH.

**Figure 17:**
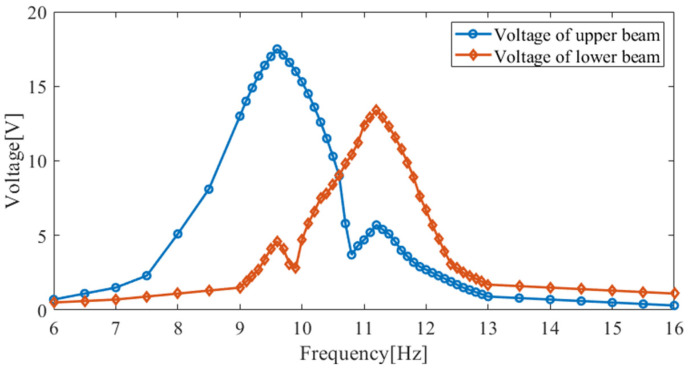
The output voltage of the HPEBH’s piezoelectric harvester.

As shown in Figure 17, the upper piezoelectric cantilever beam’s output voltage is 17.5 V (9.6 Hz), the lower piezoelectric cantilever beam’s output voltage is 4.6 V (9.6 Hz), and the total output voltage is 22.1 V (9.6 Hz). The lower piezoelectric cantilever beam’s output voltage is 13.4 V (11.2 Hz), the upper piezoelectric cantilever beam’s output voltage is 5.7 V (11.2 Hz), and the total output voltage is 19.1 V (11.2 Hz). In the simulation, the output voltage of the HPEBH’s piezoelectric harvester is 23.1 V at the first resonance point and 19.8 V at the second resonance point. Therefore, the output voltages are lower in the experiment than in the simulation. The parameters of the cantilever beams may differ slightly in the experiment and the simulation.

**Figure 18:**
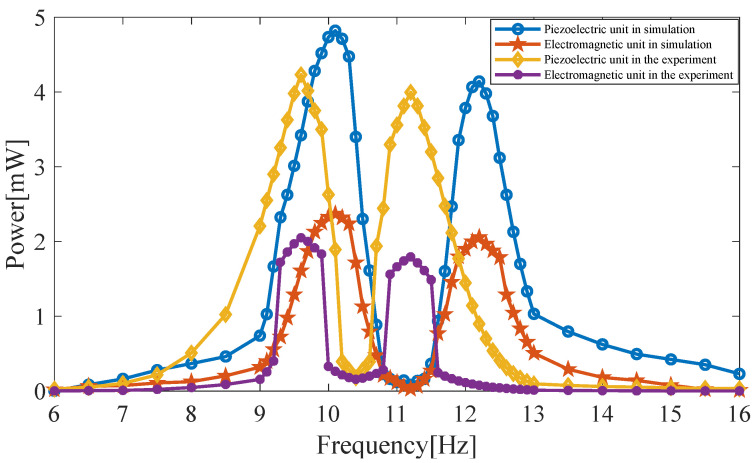
Comparison of the output power of the HPEBH obtained from the simulation and the experiment.

The optimal loads are used to calculate the HPEBH’s output power. As shown in Figure 18, at 9.6 Hz and 11.2 Hz, the total output power of the upper and lower beams of the piezoelectric harvester in the experiment is 4.23 mW and 3.99 mW, respectively, and that of the electromagnetic harvester is 2.05 mW and 1.79 mW, respectively. Therefore, the total output power of the HPEBH in the experiment is 6.28 mW at 9.6 Hz and 5.78 mW at 11.2 Hz. In the simulation, the total output power of the HPEBH is 7.19 mW at 10.1 Hz and 6.21 mW at 12.2 Hz. The output power obtained is slightly lower in the experiment than in the simulation because of the difference between the experimental equipment and an ideal experimental model.

In the test, the coil has no current and no electromagnetic damping force if the electromagnetic harvester is disconnected from its load. Thus, this test reflects the performance of the piezoelectric harvester. The piezoelectric harvester’s output power is 5.15 mW and 4.70 mW. Therefore, the total output power of the HPEBH in the experiment is 21.94% higher at 9.6 Hz and 22.98% higher at 11.2 Hz than that of the piezoelectric harvester.

The total output power of the HPEBH is much higher than that of the piezoelectric harvester or the electromagnetic harvester in the experiment; this is consistent with the simulation results. The electromagnetic damping force affects the energy-harvesting efficiency of the HPEBH. Nevertheless, the electromagnetic harvester has a high output current, which can be used as a supplement to the piezoelectric harvester. Thus, the total output power of the HPEBH and the ambient energy utilization rate are higher.

The frequency range of the two resonance peaks is much broader than the effective energy-harvesting bandwidth of the single-beam structure. The effective bandwidth of the HPEBH is in the low-frequency range (9 Hz to 13 Hz), which is consistent with the frequency band of human movement. Thus, the HPEBH has a broad application range. The frequency values at the two resonance points are slightly lower in the experiment than in the simulation, a fact that can be attributed to the slight difference in the HPEBH structure between the experiment and the simulation.

When the electromagnetic damping force acts on the surface of the permanent magnet of the HPEBH, the force is transmitted to the piezoelectric cantilever beam through the springs. The springs store and release energy, reducing the sudden impact of environmental vibration and broadening the frequency band. More importantly, it improves the durability of the piezoelectric cantilever beam; meanwhile, the upper and lower springs play a guiding role for the permanent magnet to prevent the loss of energy.

The comparison of the power-generation performances of different energy-harvesting devices is listed in Table 2.

The data in Table 2 indicate that the resonant frequency of the proposed HPEBH is lower than that of the devices described in [23,25,26,28,34] and consistent with the frequency range of human movement. The output power ranks third after the devices used in [26,35]. In addition, the proposed HPEBH has a relatively high power density.

## 5. Conclusions

A novel HPEBH with a double-beam structure was proposed. Two parallel piezoelectric cantilever beams were used to harvest ambient vibration energy based on the piezoelectric effect. A permanent magnet was connected by springs to the two piezoelectric cantilever beams. A fixed coil surrounded the moving permanent magnet to harvest electromagnetic energy. A mathematical model of the HPEBH was established. The output power of the piezoelectric harvester and electromagnetic harvester and the proposed HPEBH were obtained from a simulation. The total output power of the HPEBH was 7.19 mW at the first resonance point, which was 22.5% higher than that of the piezoelectric harvester. The total output power of the HPEBH at the second resonance point was 6.21 mW, which was 23.7% higher than that of the piezoelectric harvester. The effective energy-harvesting bandwidth was 4 Hz, higher than that of the single-beam structure and within a low-frequency band range of 9 Hz to 13 Hz, which is consistent with human movement. A HPEBH prototype was built and evaluated in an experiment. After the optimal loads were used, the total output power of the upper and lower beams of the HPEBH’s piezoelectric harvester at 9.6 Hz and 11.2 Hz was 4.23 mW and 3.99 mW, and that of the HPEBH’s electromagnetic harvester was 2.05 mW and 1.79 mW, respectively. The total output power of the HPEBH was 21.94% higher at 9.6 Hz and 22.98% higher at 11.2 Hz than that of the piezoelectric harvester. The HPEBH has two resonance points in the first-order vibration mode, resulting in a broader effective energy-harvesting bandwidth and higher output power.

## Figures and Tables

**Table 1:** Material parameters.

Material	Parameters	Values
PZT-5H	Young’s modulus	56 GPa
	Density	7500 kg/m^3^
	Poisson’s ratio	0.36
Beryllium bronze	Young’s modulus	112 GPa
	Density	8780 kg/m^3^
	Poisson’s ratio	0.35
NdFeB	BH (max)	35 MGOe

**Table 2:** Comparison of power-generation performances of various energy-harvesting devices.

Reference	Frequency (Hz)	Acceleration (g)	Power (mW)	Power Density (μW/cm^3^)
This work	10.1	0.5	7.19	44.65
Lin et al. [23]	25	1.0	2.173	85.28 *
Li et al. [24]	5.57873	1.0	5.49	27.56
Challa et al. [25]	21.6		0.332	9.5
Pyo et al. [26]	57		7.38	2.952
Foisal et al. [27]	8.5	0.5	2.09	52.02
Ganapathy et al. [28]	44		0.659	10.98
Song et al. [33]	5		2.2609	11.3
He et al. [34]	20	0.5	0.1075	0.676
Aldawood et al. [35]	11	0.4	68.78	315.2

* The device’s volume was estimated as 25.48 cm^2^ from the data provided in the article.

## Data Availability

Not applicable.
